# Novel Occlusion-Reopening Technique for Perigraft Seromas in Arteriovenous Grafts: A Multicenter Case Report

**DOI:** 10.1016/j.xkme.2026.101240

**Published:** 2026-01-05

**Authors:** Wenshen Pu, Yong Xu, Jiali Liu, Ling Chen, Qiquan Lai, Xuejing Gao, Bo Tu, Bo Hu, Ziming Wan

**Affiliations:** 1 Department of Nephrology, Baoshan People’s Hospital, Baoshan, China; 2 Department of Nephrology, The Third Xiangya Hospital of Central South University, Changsha, China; 3 Department of Clinical Medicine, North Sichuan Medical College, Nanchong, China; 4 Department of Nephrology, The First Affiliated Hospital of Chongqing Medical University, Chongqing, China; 5 Department of Ultrasonography, The First Affiliated Hospital of Chongqing Medical University, Chongqing, China; 6 Department of Nephrology, The First Affiliated Hospital of Jinan University, Guangdong, China

**Keywords:** arteriovenous graft, case report, graft occlusion, reopening technique, seroma

## Abstract

Arteriovenous grafts remain crucial for hemodialysis access despite the risk of perigraft seromas (PSs)—sterile fluid collections causing graft compression, thrombosis, and dysfunction. Current treatments (surgical drainage and compression therapy) show high recurrence rates. We describe a novel occlusion-reopening technique for refractory PSs in 5 patients receiving hemodialysis (mean age, 62.6 years) with expanded polytetrafluoroethylene AVGs. All seromas developed at arterial anastomoses (1-43 days after placement) and recurred after conventional therapies. Our protocol involved (1) intentional graft occlusion via manual compression to induce thrombosis, (2) complete surgical seroma evacuation with pseudocapsule preservation, and (3) percutaneous transluminal angioplasty using 6-mm balloons for graft reopening (see Fig 1 for details). Technical success was achieved in all cases, with immediate restoration of thrill/bruit and mean post–percutaneous transluminal angioplasty flow of 1320 mL/min. No seroma recurrences occurred during follow-up, and all AVGs remained functional. The occlusion-reopening technique addresses PS pathophysiology by eliminating fluid production through temporary thrombosis while preserving graft architecture. This approach demonstrates 100% efficacy in our series for recurrent PSs unresponsive to standard therapies, offering a reproducible solution with durable outcomes.

## Introduction

Although arteriovenous fistulas are most commonly used as vascular access routes in patients receiving hemodialysis, arteriovenous grafts (AVGs) are frequently used in cases in which an arteriovenous fistula cannot be established. A perigraft seroma (PS), often referred to as a “weeping graft”, is characterized by the accumulation of sterile fluid around the graft, accompanied by the formation of pseudocapsules, which can lead to enlargement.^1^ This condition may result in complications such as graft infection, skin necrosis, and graft thrombosis.^2^ The incidence of PS development following AVG placement for hemodialysis is approximately 1.7%^3^; however, this value may be underestimated because of spontaneous resolution of symptoms, their mild nature, and late development. Optimal treatment strategies for PS are unclear, and recurrence potential poses a significant concern because it can ultimately lead to AVG thrombosis and dysfunction. In this study, we introduce a novel occlusion-reopening technique for reducing the incidence of PS development after AVG placement.

## Case Presentation

Five patients receiving hemodialysis (1 female) were admitted to the access center for the management of AVG seromas. The etiology of kidney failure in 4 patients was chronic glomerulonephritis, whereas the remaining patient had polycystic kidney disease. None of the patients had a history of diabetes. As detailed in Table 1, the average age of the 5 patients at the time of AVG placement was 62.6 years (range, 45-86 years), with a mean duration of dialysis of 35.0 months (range, 1-78 months). The mean hemoglobin level was 100.2 g/L (range, 68-129 g/L), and the mean albumin level was 40.3 g/L (range, 34-49 g/L).

**Table 1:** Clinical Characteristics, Interventions, and Results of the Reported Cases

Patient	1	2	3	4	5
Sex	M	M	F	M	M
Causes of kidney failure	Polycystic kidney	CGN	CGN	CGN	CGN
At time of graft placement					
Age (y)	57	57	68	45	86
Dialysis duration (mo)	2	1	47	78	47
Diabetes mellitus	N	N	N	N	N
Prior vascular access	Y	Y	Y	Y	Y
Hemoglobin (g/L)	107	92	68	105	129
Serum albumin (g/L)	47	40.3	34	41	39
AVG type	Left forearm loop BB-AVG	Left forearm loop BB-AVG	Left thigh loop SF-AVG	Right forearm loop BB-AVG	Right forearm loop BB-AVG
Graft material	ePTFE	ePTFE	ePTFE	ePTFE	ePTFE
Graft size (mm)	4-6	4-6	4-6	4-6	4-7
Time from graft placement to seroma development (d)	16	43	1	25	28
Seroma position	Arterial anastomosis	Arterial anastomosis	Arterial anastomosis	Arterial anastomosis	Arterial anastomosis
Seroma size (mm)	31 × 19	28 × 20	25 × 15	24 × 18	21 × 9
Primary treatment	Surgical removal	Local compression	Surgical removal, percutaneous drainage, and PTA	Surgical removal and percutaneous drainage	Occlusion-reopen
Recurrence	Y	Y	Y	Y	N
Secondary treatment	Occlusion-reopen	Occlusion-reopen	Occlusion-reopen	Occlusion-reopen	—
Time from occlusion to graft reopening (d)	11	21	30	14	18
Time from graft puncture to reopening (d)	0	1	13	8	15
Current status of grafts	Functioning	Functioning	Functioning	Functioning	Functioning

Abbreviations: AVG, arteriovenous graft; BB-AVG, brachial–basilic arteriovenous graft; CGN, chronic glomerulonephritis; ePTFE, expanded polytetrafluoroethylene; F, female; M, male; N, no; PTA, percutaneous transluminal angioplasty; SF-AVG, superficial femoral–femoral arteriovenous graft; Y, yes.

In this study, all 5 patients underwent implantation of an AVG composed of expanded polytetrafluoroethylene. Among these patients, 3 were treated with 4-6 mm Venaflo II AVG (Bard Peripheral Vascular), 1 patient received a 4-7 mm Venaflo II AVG, and the remaining patient was implanted with a 4-6 mm PROPATEN AVG (W. L. Gore & Associates, Inc). The sizes of the AVG were 4-6 mm tapered for 4 patients and 4-7 mm tapered for 1 patient. The placement of the AVG varied among the 5 patients. Specifically, patients 1 and 2 underwent implantation of a left forearm loop brachial–basilic AVG. Patient 4 received a right forearm loop brachial‒basilic AVG, whereas patient 3 received a left thigh loop superficial femoral‒femoral AVG. Finally, patient 5 underwent implantation of a right forearm loop brachial–basilic AVG.

Seromas developed between 1 and 43 days after surgery, and all seromas were localized at the arterial anastomosis. The seromas presented as painless masses. Physical examination revealed diminished thrill and bruit in the AVG. Doppler color ultrasound confirmed the presence of a seroma around the arterial anastomosis. Patient 2 underwent conservative management with local compression. Patient 1 underwent surgical seroma removal, and patients 3 and 4 underwent surgical seroma removal as well as percutaneous drainage. Notably, Patient 3 also underwent percutaneous transluminal angioplasty with a 6 mm × 40 mm Mustang balloon (Boston Scientific) because of external compression occlusion resulting from the seroma.

Patients 1 to 4 experienced seroma recurrence following initial treatment. To minimize repeated interventions and potential complications, patient 5 underwent occlusion-reopening treatment directly. Ultimately, all 5 patients received the occlusion-reopening treatment. First, the AVGs were occluded through compression, and then the seroma was surgically excised. After 11-30 days, the AVGs were subsequently reopened during percutaneous transluminal angioplasty with 6 mm × 40 mm Mustang balloons. Upon reopening, the AVG in all 5 patients exhibited satisfactory tremor, and all were successfully punctured and used without any subsequent occurrence of seroma or hematoma.

**Figure 1:**
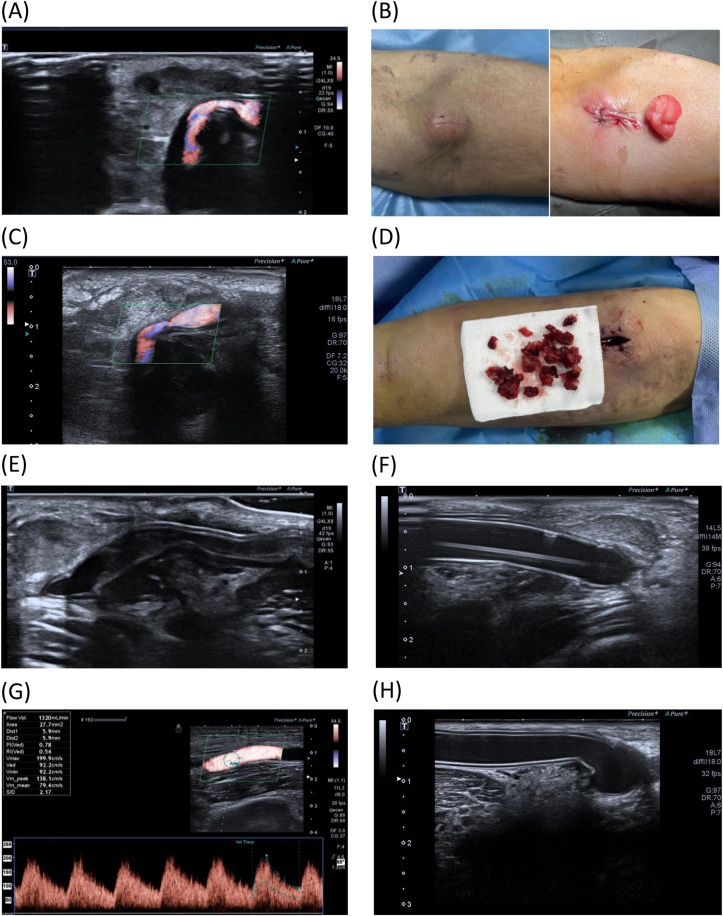
Illustrates the seroma treatment process for patient 1. (A) Doppler ultrasound showed seroma formation and graft compression 16 days after arteriovenous graft (AVG) creation. (B) Images depict the condition before and after surgical removal of the seroma. (C) Recurrence of the seroma was noted following surgical intervention. (D) The seroma was subsequently cleared again after occlusion of the AVG. (E) This image demonstrates the development of thrombosis resulting from AVG occlusion before reopening. (F) The occluded AVG was reopened through percutaneous transluminal angioplasty. (G) After percutaneous transluminal angioplasty, the AVG regained patency, and the seroma alleviated AVG compression, achieving a blood flow rate of 1320 mL/min. (H) Follow-up assessments indicated no recurrence of the seroma at the arterial anastomosis site.

**Figure 2:**
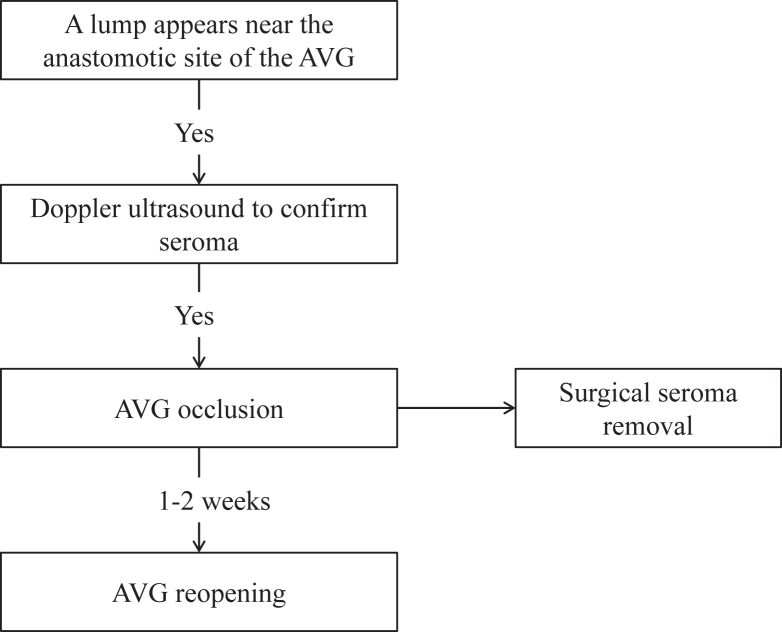
Occlusion-reopening technique for perigraft seroma in arteriovenous grafts (AVGs).

The steps of the occlusion-reopening method are illustrated in Fig 1. The steps of the occlusion-reopening method are as follows: (1) application of manual pressure to close the AVG, thus obstructing blood flow and effectively preventing continuous leakage of serum through the walls of the arteriovenous graft; (2) surgical excision of the seroma surrounding the anastomotic site of the graft to alleviate graft compression; and (3) percutaneous transluminal angioplasty for graft reopening 1-2 weeks after compression closure (see Fig 2 for flowchart).

## Discussion

In this study, we present a novel occlusion-reopening technique that can effectively treat seromas of arteriovenous graft. Currently, the mechanism of seroma occurrence is not yet clear, and the development of PS may be multifactorial, involving factors such as graft material, surgical site, surgical technique, and patient-specific characteristics.^3^^,^^4^ Previous reports indicate that PSs can manifest in various sizes and types of grafts. Doppler ultrasound is the preferred modality for evaluating graft patency and PS size.

The treatment of seroma has been primarily documented in case reports, with previous studies outlining approaches such as the watch and wait approach, bypass, evacuation, excision,^3^ covered stenting,^5^ and even graft removal in cases of recurrent PS. However, the PS recurrence rate remains high, potentially leading to AVG dysfunction. The optimal treatment for seromas remain unclear. The watch and wait approach is inefficient. Bypass surgery can effectively reduce the risk of seroma recurrence^3^ but requires a high level of surgical skill and is associated with a high risk of surgical complications, prolonged recovery, and financial burden. Graft infection may occur during evacuation treatment, leading to graft resection.^6^ Few patients are treated with covered stents because the cost is high.

Currently, in all 5 patients treated with the occlusion-reopening technique, the AVG could be used for hemodialysis without complications, as evidenced by a patency rate of 100%. The occlusion-reopening technique represents a structured approach for managing refractory PSs, developed through our multicenter experience. The protocol begins with intentional graft occlusion through controlled manual compression under ultrasound guidance, maintained until complete thrombosis is confirmed by Doppler. This initial step serves to halt ongoing serous leakage through the graft pores while preserving the structural integrity of the conduit. Within 24 hours of occlusion, surgical evacuation of the seroma fluid and associated pseudocapsule is performed, creating a controlled space between the thrombosed graft and surrounding tissues.

Following seroma evacuation, a deliberate thrombosis maintenance period of 1-2 weeks is observed. This duration was empirically determined to allow sufficient time for graft wall healing while balancing the clinical need for vascular access. During this phase, patients are monitored weekly for signs of infection or graft degeneration. The definitive reopening procedure is then performed via percutaneous transluminal angioplasty using 6-mm balloons, with technical success defined by restoration of flows exceeding 600 mL/min and absence of residual stenosis on ultrasound.

The decision to employ this protocol should follow clear criteria for conventional therapy failure, which we define as either (1) less than 50% reduction in seroma volume after 14 days of conservative management or surgical drainage or (2) development of graft dysfunction secondary to seroma compression. Post-reopening surveillance includes weekly ultrasound assessments for the first month to monitor for early recurrence, followed by standard graft surveillance protocols.

The advantages of the occlusion-reopening technique include the following: (1) lower medical and maintenance costs compared with stent implantation; (2) a potentially reduced rate of seroma recurrence, although more extensive sampling and longer follow-up periods are necessary to confirm this; and (3) a lower risk of complications as a result of early and effective treatment. However, there are notable disadvantages of the occlusion-reopening technique: (1) the indwelling time of the central venous catheter is prolonged; (2) the number of cases suitable for the technique and the duration of follow-up are insufficient, necessitating further verification of long-term hematoma recurrence rates and AVG patency rates; and (3) clinical experience suggests that AVG occlusion lasts 1-2 weeks, indicating that additional research is needed to investigate the underlying mechanism of this method and to determine the optimal occlusion duration. We hypothesize that occlusion of the AVG may activate the coagulation system, facilitating the filling of the AVG gap (the cavity between the thrombosed graft segment and the surrounding fibrotic pseudocapsule after seroma evacuation) following thrombus formation and reducing serum exudation, as indicated in the literature, suggesting that fibrin can enhance or even restore graft impermeability.^7^

## Conclusions

PSs are serious complications following graft surgery. In this study, we describe a novel treatment method used in 5 patients. Currently, all 5 patients demonstrate satisfactory patency, indicating that this method may be an effective approach for managing seromas in the future. With a mean follow-up of 8.6 months (range, 6-12 months), all 5 patients maintained graft patency. We recommend that longer-term follow-up and a larger patient cohort are essential to confirm the durability of patency.
